# Why Carbolic Acid Should Be Discarded by Dentists

**Published:** 1891-06

**Authors:** A. W. Harlan


### Why Carbolic Acid Should be Discarded by Dentists.

1. Because of its deleterious action on the pulp; oil of cloves is preferable to carbolic acid.

2. As a root canal dressing it is, because of its great solubility in water, not to be depended upon.

3. It is not a chemical disinfectant when brought in contact with sulphuretted hydrogen.

4. When it comes in contact with dentine it is absolutely useless.

5. Its beneficial action is only temporary.

6. Unless combined with oils it is valueless.

7. As an agent for injection it is without value if there are fine roots that hold fragments of pulp.

8. It does not possess embalming properties.

DR. A. W. HARLAN.

